# Toward an Affective Neuroscience Account of Financial Risk Taking

**DOI:** 10.3389/fnins.2012.00159

**Published:** 2012-11-02

**Authors:** Charlene C. Wu, Matthew D. Sacchet, Brian Knutson

**Affiliations:** ^1^Department of Psychology, Stanford UniversityStanford, CA, USA; ^2^Neurosciences Program, Stanford University School of MedicineStanford, CA, USA

**Keywords:** neuroeconomics, neurofinance, FMRI, accumbens, striatum, insula, activation likelihood estimation, meta-analysis

## Abstract

To explain human financial risk taking, economic, and finance theories typically refer to the mathematical properties of financial options, whereas psychological theories have emphasized the influence of emotion and cognition on choice. From a neuroscience perspective, choice emanates from a dynamic multicomponential process. Recent technological advances in neuroimaging have made it possible for researchers to separately visualize perceptual input, intermediate processing, and motor output. An affective neuroscience account of financial risk taking thus might illuminate affective mediators that bridge the gap between statistical input and choice output. To test this hypothesis, we conducted a quantitative meta-analysis (via activation likelihood estimate or ALE) of functional magnetic resonance imaging experiments that focused on neural responses to financial options with varying statistical moments (i.e., mean, variance, skewness). Results suggested that different statistical moments elicit both common and distinct patterns of neural activity. Across studies, high versus low mean had the highest probability of increasing ventral striatal activity, but high versus low variance had the highest probability of increasing anterior insula activity. Further, high versus low skewness had the highest probability of increasing ventral striatal activity. Since ventral striatal activity has been associated with positive aroused affect (e.g., excitement), whereas anterior insular activity has been associated with negative aroused affect (e.g., anxiety) or general arousal, these findings are consistent with the notion that statistical input influences choice output by eliciting anticipatory affect. The findings also imply that neural activity can be used to predict financial risk taking – both when it conforms to and violates traditional models of choice.

## Introduction

Imagine a world where people act as computers, consistently taking in, analyzing, and responding to all of their sensory impressions. These “rational” actors should not show volatile and inconsistent changes in preferences, and so their future choices should be predictable based on their past behavior. Such a world may be hard to imagine, because it is not the world we live in. Instead, people often show sudden, pronounced, and inconsistent changes in choice. For instance, although most people will never win the lottery or lose a limb, the same individuals will often pay a high premium both for a tiny chance to hit the jackpot as well as to compensate for the unlikely possibility of dismemberment. To explain financial risk taking, decision theorists have either appealed to the objective statistical properties of financial options or to the subjective emotional experience of individuals. Do these distinct accounts conflict with or complement each other, and can they be reconciled?

### Economic and finance models of risk taking

Traditional economic models assume that people seek to maximize value. Blaise Pascal and Pierre de Fermat historically concluded that the expected value of uncertain gambles could be calculated by multiplying the magnitude of the gamble outcomes by their probability. Thus, they mathematically defined “expected value” as the mean (or the first statistical moment) of repeated outcomes. In economics, *expected value* (and its close cousin *expected utility*) provide a foundational guide to choice by providing a common metric that individuals can use to compare different and diverse financial options (von Neumann and Morgenstern, 1944). One implication of preferences for expected value is that people should not only prefer gambles with the best outcomes, but also those with more chances to obtain a good outcome. Beyond expected value, financial theorists have additionally and separately considered the role of risk, which can be mathematically defined as variance (or the second statistical moment) of repeated outcomes (Markowitz, 1952). Resulting *mean-variance* financial models further assume that while people are attracted to expected value, they are instead repelled by risk. One implication of preferences against risk is that people should prefer gambles with relatively steady outcomes over those with more variable outcomes.

Behavioral research, however, suggests that neither expected value nor mean-variance models fully account for individuals’ financial risk taking (Edwards, 1954). As a result, some theorists have suggested that anomalies in choice (e.g., the lack of diversity in investors’ portfolios) might result from preferences for large yet improbable outcomes, which has been mathematically defined as skewness (or the third statistical moment; Mitton and Vorkink, 2007). One implication of preferences for skewness (according to some theories) is that people might prefer “long shot” gambles (e.g., those with high magnitude but low probability outcomes) over others. Despite some behavioral evidence that skewness can influence preferences (Kraus and Litzenberger, 1976; Coombs and Lehner, 1981), either by enhancing (Menezes et al., 1980) or interacting with risk (Alderfer and Bierman, 1970; Chiu, 2005), only a few models of financial risk taking consider skewness. For instance, cumulative prospect theory (Tversky and Kahneman, 1992) and rank-dependent utility models (Quiggin, 1982) have attempted to account for skewness by overweighting large but unlikely positive and negative outcomes. In doing so, however, these models sacrifice their ability to explain tolerance for variance (Levy and Levy, 2004). Although most economic theories do not account for the influence of skewed outcomes, skewed outcomes may nonetheless influence choice, at both the individual and the market levels (Patton, 2004). Thus, while traditional economic and finance theories consider the influence of mean and variance on risky choice, most remain agnostic about the influence of higher order statistical moments such as skewness. By implication, a theory that accounts for individuals’ preferences for skewness in addition to mean and variance might generate more accurate predictions about risky financial choice.

### Emotion and risky choice

If people base risky financial choices solely on statistics (e.g., mean, variance, skewness), then all individuals should show similar choices, generating predictable market movements. Psychological theorists, however, have argued that financial choices likely result from multicomponential processes that generate heterogeneous choices. If multicomponential processes drive financial risk taking, then those processes may unfold over time and be influenced by factors other than statistical moments.

Early economic theorists suspected that emotions influence choice. Smith (1759) argued that behavior was determined by a struggle between the “passions” and an “impartial spectator.” The passions included emotions such as fear and anger, as well as motivational feeling states arising from self- or other-regarding interests. Smith argued that although behavior may be influenced by passions, individuals can overcome their impulses by observing their actions from the perspective of an “impartial” outsider. Due to a subsequent emphasis on rational decision-making (partly encouraged by von Neumann and Morgenstern’s work on expected value), interest in the influence of the passions diminished.

More recently, although traditional economic theorists have endorsed the rationally grounded “Efficient Markets Hypothesis” (Samuelson, 1965; Fama, 1970), unpredicted and rapid rises and crashes of the market valuation of technology and housing sectors have raised new questions about investor rationality. Critics of the Efficient Markets Hypothesis have contended that investors consistently exhibit irrational tendencies including overconfidence (Barber and Odean, 2001; Gervais and Odean, 2001), loss aversion (Kahneman and Tversky, 1979; Shefrin and Statman, 1985; Odean, 1998), herding (Huberman and Regev, 2001), psychological accounting (Tversky and Kahneman, 1981), miscalculation of probabilities (Lichtenstein et al., 1981), and regret (Bell, 1982; Clarke et al., 1994). These “irrational” biases have been attributed to psychological factors with emotional overtones – including fear, greed, and other affective reactions to price fluctuations and shocks to wealth. In an attempt to explain individual and market anomalies, an expanding field of research has begun to examine links between emotion and “irrational” decision-making (Loewenstein, 2000).

Beyond the notion that emotion acts peripherally to undermine choice, some theorists have proposed that affect can play an even more central role by providing a “common currency” that allows individuals to compare and choose between different options (Peters et al., 2006). Despite the difficulty of measuring affect, scientists have had some success by examining associations between different affective reactions and choice (Mellers, 2000). Although most of this research has focused on “consequential” affect, which arises in response to choice outcomes, some have additionally argued for the importance of “anticipatory” affect, which occurs *prior* to choice (Loewenstein et al., 2001).

To assess affect, behavioral researchers have primarily relied upon self-reported experience. For instance, investigators can compare a single individual’s affective reactions to different stimuli (e.g., gambles) in two dimensions (e.g., valence on a continuum from bad to good, and arousal on a continuum from not aroused to aroused). Valence and arousal ratings can then be mean-deviated across stimuli within an individual and mathematically rotated through affect space (by 45°) to derive indices of positive and negative arousal (Knutson et al., 2005). Using these and related methods, investigators have shown that anticipation of uncertain monetary gains elicits positive arousal, whereas anticipation of uncertain monetary losses elicits negative arousal – even before outcomes are revealed, and when measured either online during anticipation or retrospectively (Samanez-Larkin et al., 2007; Nielsen et al., 2008).

Since anticipation of uncertain gains or losses elicits self-reported affect, this might subsequently influence risky choice. Unfortunately, anticipatory affect is difficult to assess because most affective self-reports are retrospective (and thus prone to memory and other biases) and online probes of affect may change the very nature of the choice being made (e.g., introducing reflection, distraction, delays, and other biases into the decision process). Ideally, investigators could also collect online physiological probes of anticipatory affect in order to validate and augment self-report measures. Fortunately, advances in neuroimaging at the end of the twentieth century may provide these probes.

### Neural targets

In initial attempts to link physiological measures of affect to financial risk taking, researchers collected peripheral physiological measures (including skin conductance, blood volume pulse, heart rate, muscular tone, respiration, and body temperature) from financial traders at work. The investigators observed increased physiological reactions during periods of market volatility, and reported greater increased physiological reactions to market volatility in less experienced traders (Lo and Repin, 2002). Subsequent findings suggested that the strength of physiological reactions correlated with poor trading performance (Lo et al., 2005). Contrary to the notion that emotions play no role in financial risk taking, these findings suggested that market events correlated with both self-reported and physiological reactions, even in experienced professional traders. The correlational nature of these findings, however, could not establish whether financial events caused the arousal, or whether arousal might reciprocally influence financial choice.

Advances in the temporal and spatial resolution of neuroimaging techniques (such as functional magnetic resonance imaging or FMRI) have enabled researchers to visualize changes in brain activity as individuals anticipate and make financial choices. Critically, these advances allow investigators to examine changes in neural activity in anticipation of choice. Thus, investigators can temporally capture neural responses to statistical properties of financial options before outcomes are revealed. Enhanced temporal resolution also raises the possibility of using anticipatory neural activity to predict choice. Advances in spatial resolution also matter, since FMRI allows investigators to probe activity in deep subcortical as well as cortical circuits. Based on evolutionary reasoning, while more recently evolved cortical circuits may play critical roles in the representation of language and numeric symbols, more ancient subcortical circuits that share greater homology across mammalian species may play a more prominent role in emotional and motivational functions that can promote immediate survival (MacLean, 1990). Specifically, decades of brain stimulation in animals suggest that animals will work to the exclusion of all other rewards to stimulate subcortical regions that lie along the ascending mesolimbic dopamine pathway, extending from the ventral tegmental area of the midbrain through the lateral hypothalamus to ventral striatal regions (including the nucleus accumbens or NAcc) and medial and orbital prefrontal cortices (MPFC; Olds and Fobes, 1981). In contrast, animals will work equally hard to avoid stimulating other subcortical pathways that extend from the periaqueductal gray of the midbrain up through the stria terminalis and the medial hypothalamus to the lateral amygdala, and possibly the anterior insula (Panksepp, 1998). Based on its subcortical spatial resolution, FMRI could allow investigators to test for the involvement of affect not only in choices linked to immediate survival, but also more abstract choices related to financial risk taking.

**Figure 1:**
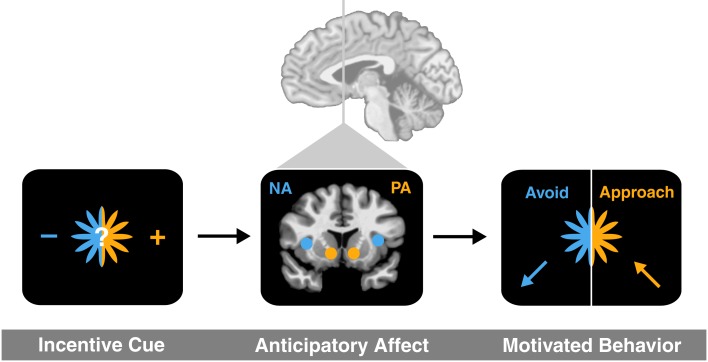
**An anticipatory affect model (adapted from Knutson and Greer, 2008)**. An incentive cue for uncertain future outcome first elicits brain activation in at least two brain regions (NAcc and anterior insula) associated with anticipatory affect (positive arousal and negative arousal, respectively). The balance of activation in related circuits then promotes either approach toward or avoidance of risk.

To link activity in these deep brain circuits to affective experience and ultimately choice, we have outlined an anticipatory affect model (Knutson and Greer, 2008). The model posits that uncertainty elicits increased aroused affect, while potential gains versus losses elicit positive versus negative affect. Since most future events are subjectively uncertain, potential gains should elicit positive arousal (e.g., feelings like excitement) as well as correlated neural activity in the NAcc, but potential losses should elicit negative arousal (e.g., feelings like anxiety) as well as correlated neural activity in the anterior insula. The anticipatory affect model has additional implications for motivated behavior, since the evolved function of positive arousal is to promote approach, whereas the function of negative arousal is to promote avoidance (Figure 1).

Most risky financial propositions (e.g., gambles, stocks) require concurrent assessment of uncertain gains and uncertain losses. According to the anticipatory affect model, if positive arousal increases, uncertain gains should appear more prominent, which should lead people to approach the risk (all else being equal). On the other hand, if negative arousal increases, uncertain losses should appear more prominent, which should lead people to avoid the risk. Consistent with this account, in an initial FMRI study that used neural activity to predict financial risk taking, anticipatory NAcc activity predicted increased financial risk taking, whereas anticipatory anterior insula activity predicted decreased financial risk taking (Kuhnen and Knutson, 2005).

Although initially inspired by a combination of animal brain stimulation (Panksepp, 1998) and human neuroimaging findings, the anticipatory affect model shares features with earlier “somatic marker” and “risk as feelings” models, both of which posit that anticipation of uncertain outcomes can generate emotional arousal (Bechara et al., 1996; Loewenstein et al., 2001). Critically, however, the anticipatory affect model does not require mediation through bodily sensations (i.e., requiring only brain activity, unlike somatic marker accounts), and specifically distinguishes anticipatory positive arousal from negative arousal (which have opposite effects on subsequent approach versus avoidance behavior, unlike the risk as feelings model). Finally, the anticipatory affect model links positive and negative arousal to activity in distinguishable neural circuits, implying that neuroimaging data could be used to directionally predict risky choice (e.g., Kuhnen and Knutson, 2005).

Different statistical moments of financial options might influence either the same or different neural circuits. The anticipatory affect model implies that distinct statistical moments should exert different but overlapping influences on affect and associated neural activity. First, financial options with high means involve large potential gains, and so should elicit positive arousal and correlated NAcc activity. Second, financial options with high variance involve both large potential losses and gains, which should elicit negative arousal and correlated anterior insula activity as well as positive arousal and correlated NAcc activity. Third, financial options with high (overall) skewness involve even larger potential losses and gains, which should elicit even more negative arousal and correlated anterior insula activity, as well as positive arousal, and correlated NAcc activity. However, positive skewness and negative skewness might have divergent impacts, since options with high positive skewness involve large potential gains, which should elicit positive arousal and correlated NAcc activity, while options with high negative skewness involve large potential losses, which should elicit negative arousal and correlated anterior insula activity. By implication, since anticipatory affective circuits are especially sensitive to the best or worst potential outcomes, they may de-emphasize probability and other considerations that require simulation or integration of many potential outcomes over time (and which may rely more on prefrontal circuits such as the MPFC).

**Figure 2:**
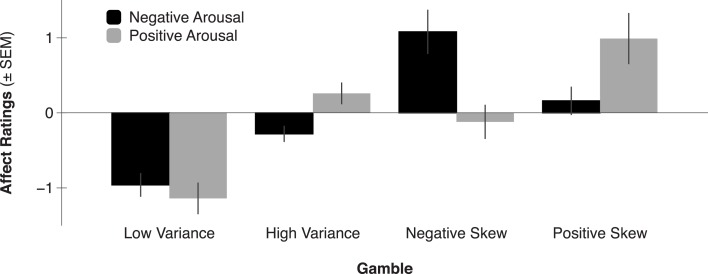
**Negative and positive arousal ratings by gamble variance and skewness (adapted from Wu et al., 2011)**. Expected value was held constant across all four gambles, while variance was equated across High Variance, Negative Skew, and Positive Skew gambles, and skewness was manipulated in opposite directions for Positive- versus Negative Skew gambles. Gambles elicited differential positive arousal such that Positive Skew > High Variance and Negative Skew > Low Variance (all *p*’s < 0.05). Gambles elicited differential negative arousal such that Negative Skew > High Variance and Positive Skew > Low Variance (all *p*’s < 0.05).

Consistent with the anticipatory affect model, previous self-reported affect findings suggest that anticipating the outcomes of higher mean gambles elicits greater positive arousal (Knutson et al., 2005). Anticipating the outcomes of higher variance gambles (with equal mean) elicits both greater negative arousal and positive arousal. Additionally, anticipating the outcomes of positively skewed gambles (with equal mean and variance) elicits greater positive arousal, whereas anticipating the outcomes of negatively skewed gambles (with equal mean and variance) elicits greater negative arousal (Figure 2; Wu et al., 2011). But beyond self-reported affect, do patterns of neural activity also align with the anticipatory affect model? Since a number of recent studies have investigated the impact of financial statistical moments on FMRI activity, we now survey their collected findings.

### Present aims

Although earlier reviews have considered how financial risk influences neural activity (Knutson and Bossaerts, 2007; Mohr et al., 2010a), none have integrated both economic and psychological accounts by explicitly linking different statistical moments of financial options to neural responses. The purpose of this meta-analysis was to examine whether different statistical moments of financial options (i.e., mean, variance, and skewness) recruit distinct or overlapping neural circuits implicated in anticipatory affect, and to explore implications of these findings for subsequent choice. To address these aims, we conducted a quantitative meta-analysis of FMRI studies of statistical moments on financial risk using the activation likelihood estimation (ALE) method (Eickhoff et al., 2011, 2012; Turkeltaub et al., 2012). Based on the anticipatory affect model, we predicted that distinct statistical moments would elicit overlapping patterns of activation, such that moments involving large gains (high mean, high variance, positive skewness) should increase activity in the ventral striatum (including the NAcc), and moments involving large losses (high variance, negative skewness) should increase activity in the anterior insula.

## Materials and Methods

### Study selection

We reviewed FMRI studies of financial risk taking. Studies were identified for meta-analysis via a search of the PubMed database using key phrases “mean” OR “reward” OR “expected value” OR “variance” OR “risk” OR “uncertainty” OR “skewness” AND “finance” OR “monetary” AND “human” AND “FMRI.” This search (performed on July 10, 2012) identified 248 studies. We specifically searched for FMRI studies that used monetary incentives to manipulate one or more of the first three statistical moments of interest (i.e., mean, variance, skewness). We further identified recent review papers about risky choice that explicitly addressed neural correlates of financial risk taking (Knutson and Bossaerts, 2007; Mohr et al., 2010a). All studies found through the database search or cited by the review papers underwent a selection process. Inclusion criteria were: (1) assessment of healthy young adults (i.e., between 18 and 60 years); (2) acquisition of whole brain FMRI data; (3) availability of peak activation coordinates from group activation tables; (4) information about the probability of uncertain outcomes was provided to participants (as opposed to ambiguity); (5) at least one of the three statistical moments of interest (i.e., mean, variance, or skewness) were objectively manipulated, independent of subjective interpretations (i.e., risk tolerance/aversion measures).

These inclusion criteria were chosen to ensure that results would generalize to the population of healthy young adult humans. Several studies suggest that aging may alter brain structure and function (Cabeza et al., 2005). Furthermore, older adults often show qualitatively different activation patterns than young adults (Park et al., 2004). Therefore, this meta-analysis focused on studies that investigated risk processing in younger healthy adults (Criterion 1). Because some studies focus on specific brain regions, they may not report whole brain results. Partial findings, however, impede the detection of unexpected activations in unscanned or unreported brain regions, so studies were excluded if they acquired or reported only partial brain data (Criterion 2). As the ALE approach requires activation foci, only studies that reported peak activation coordinates of group statistical maps were included (Criterion 3). Because risk is often conceptually distinguished from ambiguity – a form of uncertainty in which probabilities are unknown – only studies in which probabilities were known or estimated by subjects were included (Criterion 4). Since the focus of this meta-analysis was to examine neural responses to statistical moments of uncertain financial options, only studies that systematically varied mean, variance, and/or skewness (as opposed to linear probability) of monetary incentives were included (Criterion 5). Studies evaluated for variance and skewness were only included if lower order moments (e.g., mean, mean and variance) were held constant. If studies manipulated multiple moments simultaneously, the lowest appropriate manipulated moment was included in the meta-analysis (e.g., studies that manipulated variance without controlling for mean were included only for mean).

Activation maps were constructed for three distinct contrasts. For the mean map, we included contrasts of neural activity during processing of monetary incentives with high versus low mean. For the variance map, we included contrasts of neural activity during processing of monetary incentives with high versus low variance (but which controlled for mean). For the skewness map, we included contrasts of neural activity during processing of monetary incentives with high (either positive or negative) versus low skewness (but which controlled for variance and mean).

Activation foci coordinates for contrasts in the 28 studies that met inclusion criteria were submitted to ALE meta-analyses (Table 1). Of these, 21 contrasts were included in the mean map, 10 in the variance map, and 4 in the skewness map. Three studies that separately modeled mean and variance were included in both maps, and 2 studies that separately modeled variance and skewness were included in both maps. Yacubian et al. (2006) replicated their results in a second sample, thus their replication findings were separately included in the mean map. Symmonds et al. (2011) separately modeled positive skewness and negative skewness in different whole brain analyses, so these results were separately included in the skewness map.

**Table 1:** **Studies included in the ALE meta-analysis, with associated contrasts**.

Study	Mean	Variance	Skewness
Abler et al. (2009)	X		
Breiter et al. (2001)	X		
Burke and Tobler (2011)^1^			X
Christopoulos et al. (2009)		X	
Cohen et al. (2005)		X	
Delgado et al. (2008)	X		
Dreher et al. (2006)	X		
Elliott et al. (2003)	X		
Engelmann and Tamir (2009)		X	
Hsu et al. (2005)	X		
Knutson et al. (2000)	X		
Knutson et al. (2001)	X		
Knutson et al. (2003)	X		
Knutson et al. (2005)	X		
Matthews et al. (2004)		X	
Mohr et al. (2010b)	X	X	
Preuschoff et al. (2006)	X	X	
Preuschoff et al. (2008)		X	
Rademacher et al. (2010)	X		
Simon et al. (2010)	X		
Smith et al. (2009)	X		
Spreckelmeyer et al. (2009)	X		
Symmonds et al. (2011)^2^		X	X
Stoppel et al. (2011)	X		
Tobler et al. (2007)	X		
Wu et al. (2011)		X	X
Xue et al. (2009)	X	X	
Yacubian et al. (2006)^3^	X		

Total number of studies	21	10	4
Total number of foci	210	82	23
Total number of subjects	407	164	92

*^1^Whole brain coordinates acquired via personal communication*.

*^2^Modeled positive skewness and negative skewness trials separately*.

*^3^Included a separate replication sample*.

### Activation likelihood estimate rationale

In contrast to behavioral meta-analyses that aim to estimate the effect size of a finding, FMRI meta-analyses aim to identify brain regions, or circuits implicated in certain mental processes (Turkeltaub et al., 2002). Due to this difference in research goals, meta-analytic techniques have been adapted to fit the format of FMRI findings. Specifically, whereas the key results of behavioral studies are test statistics (*p*, *t*, or *z* scores) and effect sizes, test statistics in FMRI studies usually only have meaning when paired together with the information about the location of the effect, often revealed by the location of voxels with the highest test statistics. One frequently used meta-analytic technique that utilizes this spatial information is ALE analysis (Eickhoff et al., 2011, 2012; Turkeltaub et al., 2012). ALE analysis is a quantitative meta-analytic technique that compares activation likelihoods calculated from a group of observed activation foci with a null distribution of randomly generated activation foci. The ALE meta-analytic method provides advantages over traditional label-based meta-analytic methods because it relies upon activation foci coordinates, which show greater reliability across FMRI studies than do anatomical labels.

Meta-analyses were conducted using the ALE algorithm implemented with Ginger ALE software available from www.brainmap.org (Laird et al., 2005). Foci originally reported in Montreal Neurological Institute coordinates were converted to Talairach coordinates using the icbm2tal transformation prior to analysis (Lancaster et al., 2007). In the ALE analyses, each contrast’s activation foci are modeled as the peaks of Gaussian functions, the spatial extent of which is dependent on the number of subjects included in the corresponding analysis. The resulting distributions of values (called “activation likelihood estimates”) represent the probability of activation occurring in a given voxel (i.e., the ALE values). For the whole brain ALE values, significance was assessed against 5000 sets of randomly distributed foci with a non-parametric statistical permutation test. Statistically thresholded maps were then computed using a false discovery rate procedure that corrected for multiple comparisons across the whole brain [FDR (*q*) = 0.01, minimum cluster size = 100 mm^3^].

## Results

**Figure 3:**
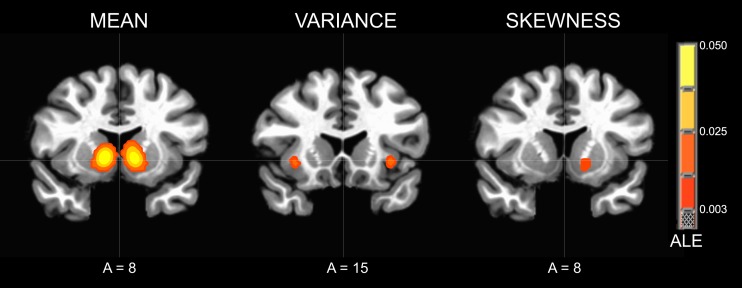
**Activation Likelihood Estimate (ALE) meta-analytic maps for high versus low mean, variance, and skewness**. ALE of mean: bilateral NAcc. ALE of variance: bilateral anterior insula. ALE of skewness: left NAcc.

The contrast of neural responses to high versus low mean (studies = 21, foci = 210, subjects = 407) had the highest probability of activating foci in the bilateral NAcc of the ventral striatum. Highly significant foci were also observed in the anterior cingulate cortex, followed by the bilateral anterior insula. Other significant foci were observed in the left red nucleus, thalamus, and putamen (Table 2; Figure 3).

**Table 2:** **ALE of neural foci implicated in processing high versus low mean, variance, and skewness**.

Region	ALE (X10^-3^)	*x*	*y*	*z*
**MEAN (HIGH VERSUS LOW)**				
**Right ventral striatum**	**55.6**	**10**	**8**	**2**
**Left ventral striatum**	**51.4**	**−10**	**6**	**2**
Left anterior cingulate	24.0	0	22	32
Right anterior insula	20.0	34	16	2
Left red nucleus	20.5	−2	−18	−12
Left anterior insula	16.7	−30	18	0
Left thalamus	16.2	0	−14	14
Left cingulate	15.0	0	2	46
Left putamen	14.2	−26	−2	4
**VARIANCE (HIGH VERSUS LOW)**				
Left subgenual cingulate	14.5	0	22	−6
**Left anterior insula**	**14.1**	**−32**	**16**	**0**
Left superior temporal cortex	13.8	−54	−10	4
**Left ventral striatum**	**13.3**	**−12**	**8**	**−2**
Right medial prefrontal cortex	12.2	2	44	30
**Right anterior insula**	**13.1**	**32**	**14**	**−2**
**SKEWNESS (HIGH VERSUS LOW)**				
**Left ventral striatum**	**11.5**	**−14**	**8**	**−2**

*(In Talairach space, *x* = right-left; *y* = anterior-posterior, and *z* = superior-inferior coordinates; predicted peak foci in **bold**)*.

The contrast of neural responses to high versus low variance (studies = 10, foci = 82, subjects = 164) had the highest probability of activating foci in the left subgenual cingulate cortex and left anterior insula. Significant foci were also observed in the left superior temporal sulcus, left medial prefrontal cortex, right ventral striatum, and right anterior insula.

The contrast of neural responses to high versus low general skewness (studies = 4, foci = 23, subjects = 92) had the highest probability of activating foci in the left NAcc of the ventral striatum.

## Discussion

This meta-analysis aimed to determine whether distinct statistical moments of risky financial options (i.e., mean, variance, skewness) elicit different patterns of neural activity. Rather than recruiting either the same or completely distinct circuits, statistical moments activated overlapping circuits implicated in anticipatory affect. Specifically, statistical moments that promised large gains (i.e., high mean, high variance, high skewness) maximally activated the ventral striatum (particularly in the NAcc), whereas moments that threatened large losses (i.e., high variance) maximally activated the anterior insula. The deep subcortical localization of these circuits is noteworthy (as opposed to neocortical structures implicated in symbolic representation and working memory), as it implies that affective rather than cognitive processes play a critical role in financial risk assessment.

Most of the findings were consistent with the anticipatory affect model. Specifically, high versus low mean maximally activated ventral striatum (including the NAcc), high versus low variance maximally activated the anterior insula (and secondarily the ventral striatum), and high versus low skew maximally activated the ventral striatum. However, high versus low mean also activated the anterior insula to a lesser extent. This may be due to the fact that while most studies of higher order moments (e.g., variance and skewness) controlled for lower order moments (e.g., mean), studies of lower order moments typically did not control for higher order moments. Because increasing lower order moments (e.g., mean) often also increases higher order moments (e.g., variance), studies of lower order moments may inadvertently elicit activity related to higher order moments. Reduced control of higher order moments in studies of lower order moments might also account for the larger overall number of activation foci observed in the high versus low mean contrast.

Findings for skewness partially conformed to the anticipatory affect model. While general skewness activated ventral striatum (including the NAcc), as predicted, common activation of the anterior insula was not as apparent. The omission is unexpected given that all three surveyed studies of skewness have reported that skewed gambles tend to activate the anterior insula (Burke and Tobler, 2011; Symmonds et al., 2011; Wu et al., 2011). The small number of relevant studies and variability of activation foci in the anterior insula may have precluded a common finding. The anticipatory affect model also specifically predicts that positively skewed gambles will more powerfully activate the ventral striatum, as was found in one study (Wu et al., 2011). However, this prediction could not be evaluated in the context of the meta-analysis because all studies did not provide contrasts for positive versus negative skewness, though this represents an important direction for future research. Finally, some studies modeled statistical moments during the uncertain anticipatory period before gambles were evaluated, whereas others modeled the entire gambling episode from anticipation to outcome. Since the anticipatory affect model is most relevant to the uncertain anticipatory period, it might best predict neural activity that occurs then.

### Integrating anticipatory affect and financial risk taking

The meta-analytic findings support neither monolithic nor modular views of neural responses to the statistical moments of financial options. Specifically, ascending from mean (lower order) to skewness (higher order moments) neither repeatedly activates all the same regions, nor does it recruit wholly distinct regions at each step. Thus, ordering the findings by objective statistical properties of the options does not yield a coherent framework for predicting associated neural activity (Table 3).

**Table 3:** **Predicted maximum activity organized by statistical moments (lower to higher order)**.

		Brain		Affect		Choice
		NAcc	Anterior insula	Positive arousal	Negative arousal
Statistics	Mean	X		X		↑
	Variance		X		X	↓
	+Skew	X		X		↑
	−Skew		X		X	↓

Alignment by affective impact, however, reconfigures the statistical moments in a coherent way that generates more consistent predictions about associated neural activity (Table 4). Specifically, financial options that involve uncertain large gains are likely to elicit positive arousal (e.g., high mean, positive skewness) and recruit NAcc activity, but financial options that involve uncertain large losses are likely to elicit negative arousal (e.g., high variance, negative skewness) and recruit anterior insula activity. Reordering these statistical moments by affective impact thus scaffolds a more parsimonious and coherent framework for predicting choice. Thus, statistical moments representing objective financial risk may be translated into subjective feelings of risk indexed by neural circuits associated with affect, which together promote choice. Of course, statistical moments may also influence choice through other neural routes as well. For instance, statistical information might recruit circuits involved in symbolic representation and working memory for numerical computation (e.g., dorsolateral and parietal cortices), or might activate circuits implicated in following habits or rules (e.g., the dorsal striatum and premotor cortex). The current analysis, however, suggests that an affective neuroscience account may provide an initial viable framework both for describing and predicting financial risk taking.

**Table 4:** **Predicted maximum activity organized by affective impact**.

		Brain		Affect		Choice
		NAcc	Anterior insula	Positive arousal	Negative arousal	
Statistics	Mean	X		X		↑
	+Skew	X		X		↑
	Variance		X		X	↓
	−Skew		X		X	↓

### Implications for financial choice

While traditional economic (e.g., expected value) and finance (e.g., mean-variance) models can account for a range of choices, other choices elude these models’ explanatory reach. For instance, individual choices may be influenced by higher order statistical moments (e.g., skewness, kurtosis) as well as by incidental factors that are not relevant to the choice at hand (e.g., news, weather, nutrition, sleep, etc.). By both encompassing and transcending the explanatory reach of traditional models, an affective neuroscience approach may eventually offer a more comprehensive account of financial risk taking.

Although this meta-analysis focused on the influence of financial options on neural activity, the anticipatory affect account also has implications for choice. Indeed, neuroimaging evidence suggests that while ventral striatal activity (and NAcc activity in particular) predicts risk seeking stock choices, anterior insula activity instead predicts risk avoidant bond choices in investment tasks (Kuhnen and Knutson, 2005). Extended to higher order statistical moments, individual differences in NAcc activation as well as positive arousal predict subsequent preferences for positively skewed gambles (Wu et al., 2011). These findings suggest that even given the same statistical gambles, individual differences in affective and neural responses may provide finer-grained predictions that describe not only group behavior, but also individual choice. These findings also imply the novel prediction that even after holding mean and variance constant, ventral striatal (NAcc) activity should predict approach toward positively skewed gambles, while anterior insula activity should predict avoidance of negatively skewed gambles – a prediction worthy of further investigation. Thus, different types of financial risk (e.g., variance versus skewness, positive versus negative skewness, etc.) may differentially recruit circuits involved in financial risk taking.

An affective neuroscience account also yields novel predictions about the influence of incidental stimuli on financial risk taking. Specifically, stimuli that increase positive arousal should encourage financial risk taking, whereas stimuli that increase negative arousal might discourage financial risk taking, even when those stimuli are irrelevant to the task at hand. Indeed, in a neuroimaging study of heterosexual males, exposure to positive images (i.e., erotic – versus neutral office supplies or aversive snakes and spiders) tended to increase choices of higher risk (i.e., higher variance) gambles, and this effect was partially mediated by NAcc activation (Knutson et al., 2008). In a follow-up behavioral study that included males and females, prior presentation of positive images increased financial risk taking, but prior presentation of negative images decreased financial risk taking (Kuhnen and Knutson, 2011).

While these influences may hold in tightly controlled and carefully incentivized laboratory demonstrations, do they generalize to “real world” choices? Researchers have speculated that investors continue to show biases in choice despite financial advice or knowledge to the contrary. Some of these, such as the lack of diversity in investment portfolios, may result from preferences for skewness (Mitton and Vorkink, 2007). Additionally, because individuals are willing to pay more for positively skewed investments but receive more for accepting negatively skewed investments (Ang et al., 2006), skewness preferences may not only describe individual investment choices, but may even scale to market valuation at the aggregate level (Arditti and Levy, 1975).

In summary, to explain human financial risk taking, economists have traditionally referred to objective statistical properties of financial options, while psychologists have emphasized subjective emotional and cognitive processes in the decision maker. An affective neuroscience account bridges these perspectives by proposing that the brain translates statistical input into affective experience, which then can influence choice. Importantly, this affective neuroscience account generates novel yet testable predictions of how various statistical moments might influence choice, and further specifies which neural components should translate statistical input into choice output. The existing findings summarizing neural responses to the first three statistical moments of financial options (i.e., mean, variance, and skewness) lends support to an affective neuroscience approach. Future work using brain activity to predict choice may generate predictions that transcend traditional economic and psychological theories. Ultimately, a better understanding of the neural mechanisms that influence financial risk taking may not only improve individuals’ financial choices, but also societal welfare by better informing public policy.

## Conflict of Interest Statement

The authors declare that the research was conducted in the absence of any commercial or financial relationships that could be construed as a potential conflict of interest.
